# Breeding an underutilized fruit crop: a long-term program for *Hylocereus*

**DOI:** 10.1093/hr/uhac078

**Published:** 2022-04-11

**Authors:** Noemi Tel-Zur

**Affiliations:** French Associates Institute for Agriculture and Biotechnology of Drylands, Blaustein Institutes for Desert Research, Sede Boqer Campus, Ben-Gurion University of the Negev, P.O.B. 653, Beer Sheva 84104000, Israel

## Abstract

This review describes three decades of introduction, agro-technology development, breeding and selection of *Hylocereus* species, known as pitaya or dragon fruit, as an example of a holistic program aimed to develop the horticultural potential of a perennial underutilized fruit crop. Interspecific homoploid and interploid crosses and embryo rescue procedures produced improved hybrids, some of which have been released to farmers. Molecular tools and morphological and phenological comparisons between the parental species and the resulting hybrids provided valuable information on dominant/recessive traits and on genetic relationships that could be exploited for further hybridizations. In addition, *Hylocereus* were crossed with species of the closely related genus *Selenicereus*, producing valuable intergeneric hybrids. In situ chromosome doubling resulted in the production of autopolyploid lines, from which an understanding of the effect of increased ploidy on fruit traits and metabolomic profiles was obtained. Gamete-derived lines were produced, adding to our biobank homozygote lines that were subsequently used for further hybridization. Spontaneous chromosome doubling occurred in haploid gamete-derived *Hylocereus monacanthus* lines and in interspecific interploid *Hylocereus megalanthus* × *H. undatus* hybrids obtained from an embryo rescue procedure, resulting in plants with double the expected ploidy. Challenging technical problems were addressed by the development of protocols for DNA isolation, flow cytometry, in situ chromosome doubling, androgenesis, gynogenesis and embryo rescue following interspecific and interploidy crosses. Current research leading to the development of genomics and molecular tools, including a draft genome of *H. undatus,* is also presented*.* Perspectives for further development of *Hylocereus* species and hybrids are discussed.

## Introduction

Selection of food crops is an ongoing process, in which plant species are evaluated for cultivation success, sustainability and profitability in a particular location. The crucial question that must be addressed is: Which species “deserve” to be exploited and developed as new high-value crops? This question has guided – and continues to guide – the long-term R&D project started in 1984 at Ben-Gurion University of the Negev (BGU), Israel, with the aim to introduce and domesticate wild fruit and nut species as new crops for the Israeli Negev desert, where conditions are often not suitable for supporting conventional agriculture [1]. In the framework of that project, about 40 food species from all over the world were introduced and evaluated under five dryland conditions that represented a range of climate, soil, and water characteristics. *Hylocereus* species – collectively known as pitaya or dragon fruit – showed excellent promise as fruit crops: even though the plants collapsed when grown outdoors, protection from radiation (under netting) and trellising provided the conditions that enabled the plants to grow well and to bear fruit in the first flowering season after planting [2]. A decision was therefore made to undertake a systematic and long-term breeding and selection program for *Hylocereus* species, with the BGU program being – to the best of our knowledge – the only such comprehensive project. This review aims to provide an overview of the BGU experience by summarizing three decades of research on the introduction, breeding, and development in *Hylocereus* species as an exotic fruit crop. The article begins with a very brief description of the genus and continues through the different stages of the BGU introduction and development program, where some stages were executed according to the original planning and others were added as more and more knowledge about the crop became available and as new agricultural problems/demands were encountered. The overview thereby provides a comprehensive picture of the state of the art about pitaya breeding and cultivation, as summarized in Table 1, and then goes on to touch in brief on the few aspects of the breeding program that remain to be done.

**Table 1:** Breeding stages and their goals in the BGU program for *Hylocereus* cultivation

**Stage**	**Strategy**	**Goal**	**Milestone**
1-	Introduction	Germplasm collection	Collection of more than 200 genotypes from 10 *Hylocereus* species
		Establishment of a live gene bank	The largest *Hylocereus* live gene bank in the world
		Development of agro-techniques	Development of plant growth and orchard management techniques
2-	Pre-breeding research	Identification of the introduced germplasm	Taxonomical identification of all the genotypes collected
		Cytological studies	Determination of the ploidy level and pollen viability in *Hylocereus* species. Chromosomal aberrations were found in *Hylocereus megalanthus*
		Self-(in)compatibility mechanisms	Self-incompatibility was found in diploid species and diploid hybrids
		Mating system	Crosses between all the *Hylocereus* species were found to be feasible and resulted in fruit and seed set. Metaxenia was reported
		Genetic relationships	Autopolyploid origin was posited for the tetraploid *H. megalanthus,* and the species was classified into the *Hylocereus* genus
		Development of laboratory protocols	Protocols for DNA isolation, in situ autopolyploidization, androgenesis and gynogenesis, embryo rescue, and flow cytometry analysis were developed
3-	Hybridization	Interspecific homoploid crosses	Interspecific diploid crosses resulted in viable diploid hybrids
		First interspecific interploid crosses	Reciprocal interspecific interploid crosses resulted in triploid, pentaploid and hexaploid hybrids
		Second interspecific and back-cross crossing cycles	Interspecific interploid crosses and back-crosses resulted in viable allopolyploid hybrids, including improved self-compatible allotetraploids
4-	Intergeneric hybridization	Intergeneric homoploid and interploid crosses	Reciprocal *Hylocereus-Selenicereus* crosses yielded viable intergeneric diploid and triploid hybrids
5-	Autopolyploidization	Artificially induced in situ genome duplication	Autotetraploid, autohexaploid and autooctaploid lines were obtained
6-	Homozygous lines	Anther and ovule morphology and development	The timing of pollen and of ovule developmental stages was determined
		Androgenesis	Homozygous lines were created via androgenesis
		Gynogenesis	Homozygous lines were created via gynogenesis
7-	Embryo rescue	Embryo development	The timing of the different embryo developmental stages was determined
		Homoploid interspecific crosses	Hybrids were obtained from embryo rescue following homoploid interspecific crosses
		Interploid interspecific crosses	Hybrids were obtained from embryo rescue following interploid interspecific crosses
8-	Further breeding and development	Breeding and selection for tolerance to extremely high temperatures	Allotetraploid cultivars with improved tolerance to high temperature were produced
		Grafting elite cultivars	Grafting produced plants with enhanced tolerance to high temperatures
9-	Nutritional value	Pigment identification/ profiling in red pulp pitaya	A novel betacyanin named “hylocerenin” was identified
		Breeding and selection for improved nutritional values	To be done
10-	Genomics and molecular genetics studies	Betalain biosynthesis	Conserved and novel miRNAs were identified Advanced knowledge in transcriptional regulation of genes associated with betalain biosynthesis was acquired. Candidate genes of the betacyanin pathway were identified and localized in the genome
		Tolerance to abiotic stresses	Identification of proteins related to cold stress Identification of putative genes and metabolites involved in heat- stress response
		Flowering process and anthesis time	Platform for studying the molecular mechanism controlling floral induction and regulation Identification of 33 aquaporin genes
		Gene editing	The first high-quality draft genome of *H. undatus*
		Molecular breeding	To be done

### Family Cactaceae, genus *Hylocereus*

Members of the Cactaceae are native to North and South America and the West Indies [3]. Importantly, cacti are exceptionally drought tolerant, primarily by virtue of a range of specific adaptations to dry conditions, i.e. spines instead of leaves, succulent shoots, and the crassulacean acid metabolism (CAM) pathway for CO_2_ fixation [3, 4]. Today, cacti are cultivated for ornamental, medicinal, fodder, cosmetic and industrial purposes and, importantly, as vegetable and fruit crops for fresh consumption [1, 5]. Within the Cactaceae, the genus *Hylocereus* belongs to the subfamily Cactoideae, tribe Hylocereeae (Britton and Rose) Buxbaum [6]. The 15 pitaya species comprising this genus [7] are widely distributed in the tropical and subtropical regions of Central America, the West Indies, and the northern areas of South America [4, 6–8]. The plants are hemi-epiphyte perennials characterized by long triangular stems that often produce aerial roots. The flowers are large, mostly white (rarely pink or red), and nocturnal, opening in the evening or at night and lasting only a single night [9]. The fruits are large, juicy and sweet and contain numerous black, small, and crispy edible seeds. The fruits may be globose, ovoid, or oblong in shape, and they have broad scales [4, 10]. The peel is spineless in all the species with the exception of the yellow pitaya, *Hylocereus megalanthus*, which has large thorns that cover the peel (these thorns are easily removed upon fruit ripening) [9, 11]. *Hylocereus* species and hybrids are currently cultivated in Vietnam, Thailand, Malaysia, Sri Lanka, China, India, Mexico, the USA, the Caribbean region, Australia, Brazil, and Israel and also in many other countries on a small scale [1, 12, 13].

### Introduction and development of agro-techniques for *Hylocereus*: The BGU program

In its early stages, the BGU breeding program aimed to collect the necessary genetic resources from around the world for establishing a large live gene bank of *Hylocereus* genotypes [9]. The collection, established in 1984, includes more than 200 genotypes from 10 taxonomically identified *Hylocereus* species [11] (Table 1). A description of the plant material collected is available on-line at: https://www.bgu.ac.il/life/Faculty/Mizrahi/index.html.

The first species cultivated in Israel were *Hylocereus monacanthus* (Lem.) Britton and Rose and *H. undatus* (Haw.) Britton and Rose, which bear summer fruits, and *H. megalanthus* (Vaup) Bauer, which bears winter fruits. The fruits of *H. monacanthus* and *H. undatus* are large (200–600 g) and red-scaled, and have a purple and red or white pulp, respectively, and those of *H. megalanthus* are smaller (80–200 g) and have a spiny yellow peel [14, 15]. The yields obtained from the first *H. monacanthus* and *H. undatus* cultivars introduced to Israel were between 30 and 35 tons/hectare, depending on the growth conditions and the age of the orchard (personal communication, Y. Mizrahi). Although the fruits of *H. monacanthus* and *H. undatus* are strikingly decorative, their taste is inferior to that of *H. megalanthus*. Nonetheless, *H. megalanthus* is no longer cultivated in Israel, due to its low yield (~ 1 to 1.2 tons/hectare) and low profitability in the country.

Since scientific and technical information about the *Hylocereus* species was extremely limited at the time of the introduction of the plant material, it was necessary to conduct the basic research that would support the breeding program and to develop agro-techniques for *Hylocereus* cultivation. The plant growth and orchard management techniques that were developed over many years of intensive effort have been described in detail in the literature and are therefore not presented here. These agro-technologies – all aimed at ensuring profitable cultivation – included post-harvest treatments, pruning methods, vegetative propagation procedures, irrigation and fertilization regimes, and hand pollination (due to a lack of natural pollinators) [9, 16, 17]. Regarding pests and diseases, it is important to note that nematodes have been identified in Israel that damage the yellow pitaya, *H. megalanthus*, which is grown in sandy soils [1]. Fungi have also been identified in several affected fruits, but the damage to the total production is almost always insignificant [1]. In tropical countries, pitaya plants are affected by many pests, including fungi, bacteria, viruses, insects, and nematodes. The lack of diseases and pests in Israel can be attributed to the semi-arid to arid climate. During the selection program, the hybrids were studied for their performances, including their shelf-life. As such, along with the selection for fruit quality, the performances of the fruits from the hybrids were studied, aiming to identify hybrids with a shelf-life of up to 26 days, which include 21 days at 10°C followed by five days at 20 °C [1].

The *H. monacanthus* and *H. undatus* cultivars that were first introduced presented significant bottlenecks for further cultivation. Consumer feedback emphasized the poor flavor and inferior fruit taste, while the growers remarked on the short harvesting season and short post-harvest shelf life; these traits guided the selection program. The subsequent – more challenging and innovative – stages of the program (Table 1) are described in detail in the following sections.

### Cytogenetics, compatibility, and genetic relationships

Proper botanical identification, i.e. of the genus and species, is a vital stage before breeding, since it can determine mate limitation and reproductive isolation. Along with the identification of the introduced *Hylocereus* germplasm, the plant material was studied cytologically [11, 15, 18] (Table 1). In Cactaceae, the base chromosome number is *x* = 11. All the *Hylocereus* species introduced in our program are diploid species, having 2*n* = 2x = 22 chromosomes, with the exception of *H. megalanthus*, which is a tetraploid species, having 2*n* = 4x = 44 chromosomes [11, 18]. Among the species introduced, *H. undatus* and *H. monacanthus* were found to produce viable pollen grains but to show partial or complete self-incompatibility, i.e. gametophytic self-incompatibility [18, 19]. We, thus, realized that for agricultural purposes, i.e. to obtain large fruits with a high percentage of viable seeds, cross pollination would be required [16, 18, 20]. *H. megalanthus* was found to produce 70–80% of viable pollen grains and to set fruit with a similar (low) seed set after both self- and cross-pollination [16, 18, 21], with the reduced pollen viability and low seed set being attributed to chromosomal aberrations [18, 22] (Table 1). We also observed a positive correlation between the number of viable seeds and fruit weight in the *Hylocereus* species [16, 18]. Finally, metaxenia, i.e. the effect of the paternal parent on fruit ripening time, fruit size, pulp and peel dry weight and number of seeds per fruit, was reported for some species [23], indicating the importance of the donor pollen in the determination of seed and fruit set and total yield (Table 1). Protocols for pollen storage were thus developed to facilitate a constant supply of pollen for agricultural and breeding purposes, especially to enable crossing of species or genotypes whose flowering seasons do not overlap [24].

The taxonomic classification of *H. megalanthus* was problematic and over the years. In 1920, Britton and Rose [6] classified this species as *Mediocactus megalanthus*, where *Mediocactus* was a new genus named on the basis of the unique morphological features of the cactus, being “medio” between the spiny peel that is reminiscent of *Selenicereus* species and the triangular stems that are similar to those in *Hylocereus*. Later, in 1953, Moran [25] placed this species in the *Selenicereus* genus, as *Selenicereus megalanthus,* but in 2003 Bauer [7] placed this species in the *Hylocereus* genus, as *H. megalanthus*. Based on the classification of Moran (which was the accepted taxonomy at the time we started the program) and on the cytological and cytogenetic observations and molecular [random amplified polymorphic DNA (RAPD)] markers obtained in our early research work, an allopolyploid origin was suggested for this species, i.e. a natural hybridization of two diploid *Hylocereus* species or between two species from the closely related genera *Hylocereus* and *Selenicereus*, followed by chromosomal doubling [15, 18, 20]. However, in our later – more accurate – molecular work based on noncoding chloroplast DNA (cpDNA) regions, nuclear ribosomal DNA (nrDNA) internal transcribed spacer (ITS) sequences, and low-copy nuclear genes, we found that *H. megalanthus* nested within the *Hylocereus* genus [26]. The lack of evidence of an allopolyploid origin suggests a taxonomically autopolyploid or perhaps a narrowly allopolyploid [26] origin, supporting Bauer’s classification [7]. Here, it is important to remember that DNA isolation was a challenging process at the early stages of the molecular studies, because *Hylocereus* tissues are abundant in polysaccharides. A DNA isolation protocol was therefore especially developed for these species [27] (which has subsequently been used for many other challenging plant species).

The next step was to determine the nuclear DNA content of the parental species by flow cytometric analysis (which we currently use to estimate ploidy in the hybrids produced in our breeding program). The mean 2C-DNA values in the diploid *Hylocereus* species and the tetraploid *H. megalanthus* were 3.99 and 8.62 pg, respectively, supporting the ploidy already published for these species [11]. Similarly to the above-described challenge for DNA isolation, sample preparation for flow cytometry was problematic, since the polysaccharides co-precipitate with nucleic acids during the nuclear isolation procedure; thus, an improved protocol for sample preparation was also developed for this procedure [28].

### First stages of breeding – Interspecific homoploid and interploid hybridizations

**Figure 1:**
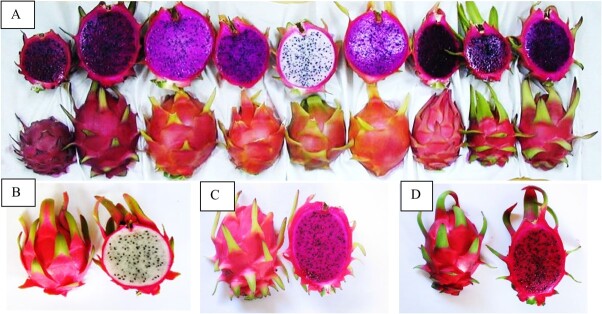
**Fruit diversity in homoploid diploid hybrids.** The homoploid diploid crosses resulted in hybrids with a high fertility level. **A.** Assorted hybrid fruits from different cross combinations. **B.** Intraspecific hybrid S-107 (*H. undatus* × *H. undatus*). **C.** Interspecific hybrid J-21 (*Hylocereus monacanthus* × *H. undatus*). **D.** Interspecific hybrid 34–07 (*H. monacanthus* × *H. undatus*). Photographs taken by Yosef Mizrahi.

Plant hybridization, i.e. mating parents from two different species or genera, has played a major role in breeding programs. Conventional plant breeding by crossing parents with specific valuable traits has produced new hybrid food crops with dramatically increased yields [29]. The initial aims of the BGU long-term breeding program were to improve fruit quality and yield and to prolong the harvesting season [9, 15, 30]. Importantly, we found that there were no barriers limiting interspecific homoploid crosses (2*n* × 2*n*) to produce diploid hybrids (Table 1). These hybrids, like their parents, showed normal meiosis, a high percentage of viable seeds, and a high germination rate. The hybrid fruits exhibited morphological traits intermediate between those of the parent species [15, 31] (Fig. 1). Morphological and phenological comparisons between the parental lines and the hybrids revealed the dominance relations that control important traits such as fruit weight, the presence of spines, flowering time, and others. The obtained information was used for further selection (See Tel-Zur et al. 2004 [15]). Our findings thus indicated that despite their taxonomic classification as different species, all the diploid *Hylocereus* species may be regarded as belonging to a primary gene pool (GP-1), since crossing and gene transfer are facile and the hybrids are fertile [32].

In addition, successful reciprocal interspecific interploidy (2*n* × 4*n*) crosses were performed between the diploid species *H. undatus* or *H. monacanthus* and the tetraploid *H. megalanthus* [15, 22, 23] (Table 1). These crosses yielded not only the expected triploid hybrids, but also pentaploid, hexaploid and aneuploid hybrids [15, 22]. More specifically, crosses between *H. megalanthus* as the female parent and *H. undatus* or *H. monacanthus* as the male parent did not yield triploid hybrids, but instead produced pentaploid, hexaploid and 6x-aneuploid hybrids. The reciprocal cross, using *H. monacanthus* as the female parent, yielded triploid and 3x-aneuploid hybrids [15, 22]. These results show the importance of the cross direction in the formation and ploidy of the resulting hybrids.

Previous work indicating the presence of unreduced gametes in *H. megalanthus* [22] suggested that the pentaploid hybrids probably originated from a fertilization event between an unreduced (4*n*) egg cell [22, 34] from *H. megalanthus* and a normal haploid (*n*) pollen grain from the diploid paternal species [22]. The origin of the hexaploid hybrids was further studied using hybrids from an embryo rescue procedure following the crossing of *H. megalanthus* with *H. undatus* [35]. The findings indicated that these hexaploid hybrids were a result of hybridization followed by chromosome doubling at the very early stages of embryo development; see below the section, *Embryo rescue following homoploid and interploid interspecific crosses.*

For breeding purposes, the most important output of this reciprocal interspecific interploidy crossing cycle was the creation of triploid hybrids whose fruits combine the attractive appearance of the maternal *H. monacanthus* fruits, i.e. medium-large fruits with red-violet pulp and peel, with the excellent taste and spiny peel of the paternal *H. megalanthus* [15]. Interestingly, the fruits of these triploid hybrids mature during the autumn, namely, they exhibit an intermediate trait between the summer and winter harvesting seasons of the maternal and paternal species, respectively. In later work, an evaluation of the breeding potential of the triploid hybrids revealed partial fertility in all the studied hybrids [33], in contrast to the complete sterility frequently observed in triploids. Since different proportions of functional female and male gametes were produced by the various hybrids, wide ranges of percentage of fruit set and viable seeds were observed [33]. Among the triploids studied, two hybrids (designated S-75 and 12–14) that were judged superior, in terms of fruit quality and yields, were released to farmers.

**Figure 2:**
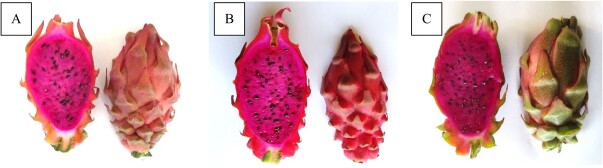
**Mature fruit from hybrids obtained following interploid interspecific crosses [S-75 (*Hylocereus monacanthus* × *Hylocereus megalanthus*) × *H. undatus*]. A.** Hybrid NB-02. **B.** Hybrid NB-52. **C.** Hybrid NB-108. Of note, there are very few thorns at the base of the peel and all the fruits set numerous viable seeds. Photographs taken by Joseph Mouyal.

A second crossing cycle was performed with the triploid hybrid S-75 as the maternal line and *H. undatus* as the paternal species. S-75 was also self-pollinated and backcrossed with *H. monacanthus* and *H. megalanthus* [36]. Several of the resulting hybrids yielded attractive and large fruits with excellent quality (Fig. 2, Table 1). Among them, the hybrids obtained from the S-75 × *H. undatus* cross (which combines three genetically and taxonomically distinct species, i.e. *H. monacanthus*, *H. megalanthus* and *H. undatus*) yielded several self-compatible tetraploid hybrids that produced a high percentage of viable pollen grains and viable seeds and excellent-quality fruits that were similar in weight following both cross- and self-pollination [36]. The tetraploidy of these hybrids can be explained as a fertilization event between an unreduced (3*n*) egg cell [34] from the S-75 triploid hybrid and a normal haploid (*n*) pollen grain from *H. undatus*. Since hybridization is indeed possible and yields hybrids with same degree of fertility, these types of crosses may be described as crosses between primary (GP-1) and secondary (GP-2) gene pools, according to Harlan and de Wet [32].

### Intergeneric hybridization

Species of the genus *Selenicereus*, which, like *Hylocereus*, belongs to the tribe Hylocereeae, grow indigenously in geographic overlap with species of *Hylocereus*. The main morphological difference that distinguishes between the two genera is the stem shape—triangular in *Hylocereus* and stellar in *Selenicereus* [4, 10]. *Selenicereus* species – like those of *Hylocereus* – also bear attractive edible fruits, but they are spiny and of poor quality*.* Controlled intergeneric crosses between species of these two genera [37] produced true hybrids (Table 1), e.g. homoploid crosses between the diploid *H. monacanthus* or *H. undatus* as the maternal parent and the diploid *S. grandiflorus* as the paternal parent produced diploid hybrids. Intergeneric interploidy crosses between *S. grandiflorus* as the maternal parent and *H. megalanthus* as the paternal parent produced genuine triploid hybrids. Morphological comparison of fruit traits between the parental species and the offspring clearly confirmed hybrid origin [37]. The formation of viable intergeneric homoploid and interploid hybrids with some viable seeds indicates a lack of post-zygote barriers between the studied species and genera [32], which is in line with the findings of a recent phylogenetic study in Hylocereeae [38]. Natural and artificial intergeneric hybrids have also been reported in other genera of the Cactaceae [39, 40] demonstrating reproductive compatibility in the genera of this family, as was found for *Hylocereus* and *Selenicereus*. The close genetic relationship between the two genera allows gene flow and the development of novel and unique plant material for breeding purposes, with the resulting intergeneric hybrids offering a possible bridge for the introgression of desirable traits.

### Autopolyploidization

Whole genome duplication might lead to morphological and/or metabolomic changes in the resulting autopolyploid plants [41], which may confer specific agricultural advantages, such as larger fruits, in comparison with the donor plant. Autopolyploids may originate via the failure of cytokinesis during a mitotic cell cycle, thus doubling the chromosome number in somatic cells, or from meiotic polyploidization, i.e. 2*n* gametes [42]. In our hands, the first successful induction of in situ autopolyploidization in Cactaceae was achieved by applying antimitotic agents to the vegetative buds and germinating seeds of a number of different species or hybrids, namely, the diploid *H. monacanthus*, the interspecific triploid hybrid designated S-75, and the tetraploid *H. megalanthus*, to produce autotetraploid, autohexaploid and autooctaploid lines, respectively [43] (Table 1). We then evaluated the potential for breeding and cultivation purposes of these autopolyploid lines [19]. An investigation of the morphological differences observed between the donor plant and its autopolyploid line revealed significant reductions in stomatal density, fruit size, number of viable seeds and pollen viability, along with an increase in pollen size [19, 43, 44]. A metabolic study of the autopolyploid lines showed that the pulp of the autotetraploid and autohexaploid fruits contained lower levels of sugars and betacyanins but higher concentrations of amino acids, tricarboxylic acid (TCA) cycle intermediates, organic acids and flavonoids vs. their donor plants [44]. From a horticultural point of view, the smaller fruits produced by the all the *Hylocereus* autopolyploids, vs. the donor plants [19, 44], indicated that increased ploidy results in decreased fitness in terms of fruit size and weight. Similar outcomes have been reported for kiwi [45] and cucumber [46], indicating that higher ploidy levels (obtained via autopolyploidization) are not necessarily associated with larger fruits, as was found in tomatillo [47] and carambola [48]. It may thus be concluded that the response to autopolyploidization in terms of fruit quality and size is species specific and depends on the genetic background of the donor species rather than on the process of genome doubling per se.

From the above it may be concluded that autopolyploidization is not a suitable breeding method to induce the production of large fruits in*Hylocereus*. Nonetheless, and importantly for breeding purposes, we judged that autopolyploidization could possibly be exploited to disrupt the self-incompatibility of *Hylocereus* species. At that time, there was no empirical evidence for a relationship between polyploidy and self-compatibility at the level of species and families [49], but evidence has subsequently accumulated in Solanaceae regarding diversification patterns and speciation related to a single event of whole genome duplication that broke down the self-incompatibility system, i.e. a one-step pathway to self compatible polyploids [50]. It was also shown that polyploidization disrupts the self-incompatibility system in apple [51] and in *Petunia hybrida* [52], among others. We did indeed find that polyploidization by a “one-step pathway” yielded a valuable horticultural improvement in that it broke down the gametophytic self-incompatibility system in the autotetraploid lines obtained from the diploid *H. monacanthus* [16, 18, 19]. The fact that the autotetraploid lines bear fruits following self-pollination offers farmers a viable alternative to hand cross pollination (albeit at the expense of fruit size), allowing the design of single-cultivar orchards.

### Creation of homozygous lines via androgenesis and gynogenesis

Haploid and double-haploid techniques are important tools for the creation of homozygous lines. These “pure lines” are extremely valuable as the maternal or paternal parent lines in plant breeding programs, because they frequently show novel genetic combinations that may be exploited for further crosses aimed to obtain improved F_1_ lines [53, 54]. Techniques for producing haploids have been known for many years and have been applied successfully for many fruit crops [55]. These techniques are based on androgenesis or gynogenesis—the process of arresting the development of normal pollen grains or egg cells, respectively, thereby inducing a shift into a somatic pathway via direct embryogenesis or via callus formation [54, 56, 57]. As a rule, plants need 6–10 generations of self-pollination to become homozygous, which is not feasible in practice in the breeding of perennial species with a prolonged juvenile phase, such as *Hylocereus*. Furthermore, for most of the diploid *Hylocereus* species, which have a strong self-incompatibility system, the use of haploid techniques is the only practical way to obtain homozygous lines.

Our efforts to develop haploid protocols started with a study of anther and ovule morphology and development in *H. monacanthus*, *H. undatus* and *H. megalanthus* [58]. We note that *Hylocereus* species have large hermaphrodite flowers, with huge numbers of megaspores and microspores per flower and a prolonged flowering season, all of which facilitate the development of haploid protocols. Androgenesis and gynogenesis protocols were successfully developed, and haploid lines were produced via direct embryogenesis in *H. monacanthus* and via direct and indirect (callus formation followed by regeneration) embryogenesis in *H. megalanthus* [59, 60] (Table 1). These were the first gamete-derived lines to be produced and reported in Cactaceae. A subsequent study using flow cytometry and simple sequence repeat (SSR) markers confirmed the gamete origin of these lines [61]. Interestingly, the five haploid *H. monacanthus* lines obtained from anther culture [60] underwent spontaneous genome doubling, resulting in double-haploid (2*x*) lines [61]. These lines showed inferior vigor to the donor line and developed flowers with an abnormal morphology, which aborted before anthesis, with the exception of a single flower that set fruit with several viable seeds. These seeds yielded eight plants that developed normally and set fruit with similar fruit morphology to that of the donor line. Thus, up to the time of writing, the double-haploid *H. monacanthus* lines and their hybrids have not added important genetic value to the breeding program.

**Figure 3:**
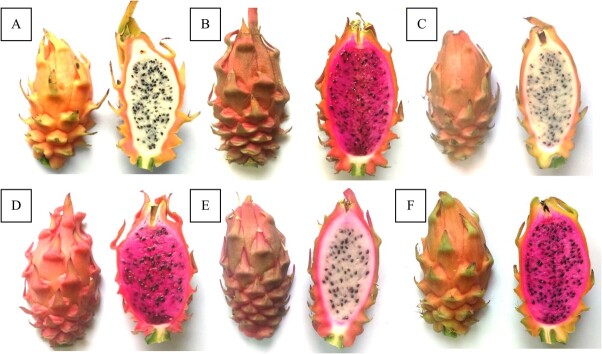
**Mature fruit from several hybrids resulting from crosses between the di-haploid *Hylocereus megalanthus* as the maternal line and *Hylocereus monacanthus* or *H. undatus* as the paternal species**. **A-F** Each show the peel and pulp of a different fruit. The fruits exhibit a variety of shapes and of peel and pulp colors, and all bear numerous viable seeds. The few thorns in the base of the peel can be easily removed at full maturation. Photographs taken by Joseph Mouyal.

The gamete-derived origin of 49 *H. megalanthus* lines obtained in this part of the breeding program was confirmed by flow cytometry and SSR markers [61]. Among the 49 lines, 46 were haploids (=di-haploids, 2*x*, since the donor plant is tetraploid) and three were double-di-haploid (4*x*) lines. Two pathways were suggested to explain the origin of the double-di-haploid (4*x*) *H. megalanthus* lines—either a normal reduced gamete (2*x*) that underwent spontaneous genome doubling or an unreduced (4*x*) gamete, as had previously been reported for this species [18]. The flowers of all the above 49 lines were smaller than those produced by the donor *H. megalanthus*, and most of them aborted at anthesis or very soon thereafter. A few flowers from the di-haploid and double-di-haploid lines set small fruit of similar weights, which were significantly lower than the average fruit weight for the tetraploid *H. megalanthus* donor plant [61]. Several hybrids were produced from the di-haploid (2*x*) or double-di-haploid (4*x*) *H. megalanthus* as the maternal line and diploid *Hylocereus* species as the parental line. The intermediate morphology of the fruits produced by these lines (J. Mouyal, personal communication) supports the hybrid origin of the plants. A preliminary (unpublished) study of these hybrids showed that some of them bear large fruits with a spiny peel, a novel (orange-pink) peel color, and an excellent taste, but pollen viability is low (Fig. 3).

Haploid lines are sterile, but following chromosome doubling fertility can be restored [53, 55]. The double-haploid *H. monacanthus* and the di-haploid and double-di-haploid *H. megalanthus* lines exhibited low or total sterility, emphasizing the strong relationship between reduced heterozygosity and low viability. The fact that an increased level of homozygosity reduced fertility (as manifested by reduced fruit and seed set) in *Hylocereus* supports the theory of “homozygote depression” [61, 62], i.e. of lower fertility in homozygote lines in comparison with their donor lines.

### Embryo rescue following homoploid and interploid interspecific crosses

Embryo rescue is a culture procedure that has been in use for many decades in breeding programs to produce living plants from undeveloped or weak embryos (mostly following hybridization), i.e. to nurture embryos that would otherwise abort. To develop an embryo rescue protocol for *Hylocereus* species, it was first necessary to have in hand information regarding the times from anthesis to the different embryo maturation stages and regarding the feasibility of technical manipulations. To this end, embryo development was studied in the different *Hylocereus* species, from fertility to maturity, to obtain data on the times from anthesis to the globular, heart, torpedo and cotyledon stages [58] (Table 1). In *H. monacanthus* and *H. undatus*, it was found that the embryo reaches the final stage, i.e. the cotyledon, 17 days after pollination (DAP), while in *H. megalanthus* 70 days are needed. Importantly, it was also found that the number of ovules per ovary is very high in *Hylocereus* species, for example, ~7200, 5300 and 2000 ovules per flower in *H. undatus*, *H. monacanthus* and *H. megalanthus*, respectively [16, 33], thus potentially providing a huge number of pollinated ovules, i.e. embryos, per flower, which makes the technical work quite feasible.

In the first stage of the embryo rescue part of the breeding program, the procedure was performed following homoploid (diploid) interspecific crosses 5 DAP, when the embryos were at very early pro-embryonic stages [63] (Table 1). The hybrid origin of the resulting plants was confirmed by fluorescent amplified-fragment length polymorphism (fAFLP), and their ploidy level was determined by flow cytometric analysis. The report of this procedure was the first describing a successful embryo rescue in Cactaceae, thus expanding the prospects for breeding programs.

In the second stage, embryo rescue was performed following interploidy interspecific crosses, i.e. *H. megalanthus* × *H. monacanthus/H. undatus* [64]. Here, too, the rescued embryos were at a very early stage of development, 10 or 30 DAP, i.e. proembryo and globular stages, respectively [58] (Table 1). Some embryos developed into normal plantlets, but others only developed callus (which did not form embryonic structures) and died. The resulting putative hybrids showed triploid, tetraploid and higher ploidy levels, and fAFLP proved their hybrid origin [64]. Fruit morphology of the true hybrids combined the traits of the two parents, but fruit weight was statistically significantly lower than that of the parents, with the hexaploid hybrids bearing smaller fruit than the pentaploid hybrids [35].

A group of putative hybrids produced by the embryo rescue technique for the *H. megalanthus* × *H. undatus* cross were further studied with the aim of isolating triploid (instead of pentaploid, hexaploid, and 5*x* and 6-*x* aneuploid [35]) hybrids that had not been obtained in our early work from germinated viable seeds following the same cross [15, 22]. Contrary to expectations, embryos isolated 10 DAP (at the pro-embryo stage) by the embryo rescue technique did not yield triploid hybrids, but rather, once again, pentaploids, hexaploids, and 5*x* and 6-*x* aneuploids [35], as were obtained from viable seeds, as described above. The pentaploids were probably a result of a fertilization event between an unreduced (2*n*) female gamete from *H. megalanthus* and a normal reduced (*n*) gamete from *H. undatus*, as was obtained following interspecific crosses (see above, the section *First stages of breeding – interspecific homoploid and interploid hybridizations*). The hexaploids and 6-*x* aneuploids obtained following embryo rescue support the above empirical evidence (see *First stages of breeding – interspecific homoploid and interploid hybridizations*) of “hybridization followed by chromosomal doubling” at the very early stages of the embryo development [35].

### Further breeding improvement: Current work

In the current era of global warming, an important aspect of our breeding program was to develop *Hylocereus* hybrids capable of withstanding heat stress. Since *Hylocereus* species originate in temperate areas, it was not surprising that high temperatures were found to inhibit flower development in *H. undatus* and *H. monacanthus* [65] and that stem damage occurred in *H. undatus* at temperatures exceeding 45°C [66]. As part of our current ongoing work, selection for tolerance to extremely high temperatures (45/35°C day/night) was undertaken by studying and comparing parental species with new allotetraploid hybrids that had been selected for their excellent fruit quality and self-compatibility [36, 67]. This comparative study showed that the parental *H. megalanthus* is the most heat sensitive species, followed by *H. monacanthus* and *H. undatus*. One allotetraploid (Z-16) showed good tolerance to extreme temperatures, and the others showed moderate to low tolerance [67], indicating that most of the hybrids performed better that their parental species and that some are indeed suitable for cultivation in heat-challenging areas.

In an experiment aimed to further improve tolerance to heat stress, the diploid *H. undatus*, the tetraploid *H. megalanthus*, a gamete-derived di-haploid *H. megalanthus* line, and a self-compatible allotetraploid (Z-10) were grafted onto *H. undatus*, and the grafted plants were compared with non-grafted plants [68]. In that work, physiological and biochemical determinations and evaluation of the expression of genes encoding heat-shock proteins (HSPs) showed that self-grafted and grafted plants recovered faster and suffered less stem damage than the non-grafted plants, suggesting that the grafting process per se induces “cross-tolerance” (plants exhibit improved performances when exposed to more than one stressor). This notion was indeed supported by the higher baseline levels of *HSP70* and *HSP90* observed in all the grafted plants under non-stress conditions, a finding that suggests a probable contribution of the grafting process to increasing stress tolerance, as manifested in a faster recovery following the stress. This work thus paves the way for combining elite allotetraploid cultivars and grafting procedures to facilitate the cultivation of pitayas under heat-stress conditions [68].

### Nutritional and health value of *Hylocereus*

Alongside breeding and genetic improvement, a start was made on studying the nutritional value of the fruits, including the determination of the fruits’ nutritional profile and the potential health benefits. The vibrant red color of pitaya fruits is attributed to the high level of biosynthesis and accumulation of the health-promoting and disease-preventing betalain pigments [3]. Among these compounds, betanin, phyllocactin and a new betacyanin named “hylocerenin” were detected for the first time in the pulp of *H. monacanthus* [69, 70] (Table 1). Other compounds identified in high levels in the peel and pulp of *Hylocereus* species included phenols, flavonoids and antioxidants [71].

Additional molecular and genomics efforts devoted to improving the nutritional value and other desirable traits are in intensive progress. In this way, we will be able to expand the uses of *Hylocereus* species and hybrids for the food and biotechnology industries.

### Advances in molecular and genomics research: Perspectives for pitaya improvement

As is the case for most underutilized crops, molecular and genomics research on *Hylocereus* species is lagging behind that on staple crops. Nonetheless, enormous progress has been made in the past few years, and any review on *Hylocereus* would not be complete without a description of this research. The basic platform for molecular and genomic studies aimed at closing the gap between phenotypes and DNA sequences was established at the South China Agricultural University in Guangzhou, China. In the first stage, the expression stability of different potential pitaya reference genes for qRT-PCR (including different tissues, growing conditions, developmental stages, etc.) was investigated and validated, providing the essential reference genes needed to calibrate the experimental conditions [72]. Advances in molecular, proteomic, transcriptomic and genomic knowledge are described below. Importantly, efforts toward sequencing the first high-quality draft genome of *H. undatus* (red peel and white pulp) were recently described by Chen et al. [73] and Zheng et al. [74]. The availability of this chromosome-level genome will open the way to further crop improvement by enabling mapping to a reference genome; instead of mapping RNA-seq reads to de novo assembled transcripts (Table 1).

#### Elucidation of betalain biosynthesis

Considerable efforts have been invested and significant progress has been made in the research on betalain biosynthesis in *Hylocereus* species. Key proteins/enzymes and genes were studied in *H. monacanthus* at white (immature) and red (mature) pulp developmental stages [75, 76]. In particular, initial insight into the molecular mechanism of betalain biosynthesis was gained through the identification of proteins differentially expressed between the white and the red pulp stages, e.g. polyphenol oxidase, CYP76AD3 and a 4,5-dihydroxy-phenylalanine (DOPA) dioxygenase extradiol-like protein. RNA-seq was then used to identify, at the transcriptome level, key genes related to betalain biosynthesis in the pulp of *H. monacanthus* by constructing two cDNA libraries of pitaya pulp, one at the immature (white) stage and the second at full maturation (red) [77]. The transcriptome data obtained was further analyzed using qRT-PCR by comparing pulp from mature fruits in *H. monacanthus* (red pulp) and *H. undatus* (white pulp), yielding several transcripts putatively involved in betalain biosynthesis in *Hylocereus* [77].

Work then turned to the WRKY protein family, a group of transcription factors (TFs) that perform a variety of cellular functions. In this context, the transcriptional regulation of genes putatively related to betalain biosynthesis, such as cytochrome P450-like (*CytP450-like*) and *HpWRKY44*, was studied [78]. The elevated expression of these two genes that was observed during the development of color in pitaya fruit corresponded to the production of higher fruit betalain levels, providing for the first time valuable insight into the transcriptional regulation of genes associated with betalain biosynthesis in *Hylocereus* (Table 1).

In addition, transcriptome and sRNAome studies were performed to explore the regulatory functions of microRNAs (miRNAs) and their target genes in betalain biosynthesis [79]. Mechanisms controlling the development of color at different developmental stages were studied, resulting in the identification of both conserved and novel miRNAs in *H. monacanthus* (Table 1). Among these miRNAs, six were found to play important roles the development of color and the accumulation of betalains in pitaya fruit. Subsequently, myeloblastosis (MYB) TFs were screened as candidate genes for betalain biosynthesis by using the genome-wide identification and characterization of *R2R3-MYB* genes [80]. In that study, 8 positive regulators and 27 negative regulators were identified by expression profiles and phylogenetic analyses, providing indications that *HuMYB1* is a potential repressor of betalain biosynthesis during pitaya fruit maturation [80] (Table 1).

In parallel to the above studies, genes of the betacyanin (a red-violet pigment present in high levels in pitaya fruits) biosynthesis pathway were identified and localized in a 12-Mb region of the chromosome 3 [74] (Table 1). The acquired data revealed the structural genes and TFs involved in betalain biosynthesis, resulting in the elaboration of a putative mechanism for betalain biosynthesis, including putative candidate genes. In addition, a high-density genetic map was constructed with the parental lines (*H. monacanthus* and *H. undatus*) and their F_1_, providing a putative gene regulatory network, including the structural genes and TFs responsible for the biosynthesis of betalains in *Hylocereus* [73].

#### Molecular studies provide initial information on tolerance to abiotic stresses

Transcripts involved in the response to cold stress were studied in pitaya plants exposed to cold (0°C) and control (23–28°C) conditions [81]. Proteogenomic studies identified both up-regulated and down-regulated proteins related mainly to chloroplast and mitochondrial metabolism, suggesting that they play an important role in the response to cold stress [81] (Table 1). In addition, transcriptomic and metabolomic studies on heat-stressed pitaya seedlings revealed numerous genes and metabolites involved in the response to heat stress, suggesting a complex stress-response mechanism [82].

#### Initial insights into the flowering process

The molecular and genetic mechanisms of pitaya flowering are largely unknown, but a start has been made in elucidating them. Xiong et al. [83] studied the molecular mechanism of flower induction by exposing plants to supplementary light, thereby revealing a significant number of differentially expressed genes. Ye et al. [84] investigated the mechanisms of pitaya anthesis, which occurs at sunset. They conducted a genome-wide analysis of aquaporins (AQPs) – membrane proteins involved in the transport of water and in the regulation of many processes, including flower opening – in *H. undatus* [84]*.* A total of 33 *HuAQP* genes distributed in 9 chromosomes were identified (Table 1). Among these, a novel AQP gene *HuNIP6;1* was suggested to play a key role in the regulation of anthesis on the basis of its expression profile [84]. These transcriptomic and genome-wide analyses have thus supplied a platform for a better understanding of the molecular mechanisms controlling the induction and regulation of flowering and of anthesis time determination in *Hylocereus* (Table 1).

## Conclusions

Three decades of *Hylocereus* research and breeding have yielded important novel biological and horticultural knowledge. Importantly, lessons learned can now be efficiently applied in the development of other underutilized crop species. The highlights of the breeding program and the current state of the art in molecular and genomics research may be summarized as follows:

The self-compatibility observed in the wild tetraploid *H. megalanthus* was also observed in the induced autotetraploid *H. monacanthus* and in artificial allotetraploids.

Spontaneous chromosome doubling was observed following interploidy interspecific crosses and in allopolyploid hybrids from the embryo-rescue technique, resulting in allohexaploidy. Spontaneous chromosome doubling was also observed in the gamete-derived haploid *H. monacanthus* (resulting in a double-haploid).

Di-haploid and double di-haploid *H. megalanthus* showed identical plant morphology to the donor species, except for flower and fruit sizes, which were significantly smaller. Gamete-derived lines showed low fertility, which was attributed to “homozygote depression.” No trait segregation was observed in the gamete-derived plants, supporting the autotetraploid origin of *H. megalanthus*. These results are in line with the molecular study using noncoding cpDNA regions, nrDNA ITS and low-copy nuclear genes.

Close genetic relationships were observed among some species of *Hylocereus* and *Selenicereus*, which enabled the production of interspecific and intergeneric hybrids with different levels of fertility. Among the interploid interspecific *Hylocereus* crosses, the cross direction determined the ploidy of the hybrid.

Increased ploidy in both autoploids and allopolyploids resulted in smaller fruits. Several self-compatible allotetraploids showed improved fruit quality and better tolerance to extremely high temperatures.

In addition to the identification of a novel betacyanin pigment named “hylocerenin,” important progress has been made in the elucidation of the biosynthesis of betalains.

Pioneer research has provided initial insights into tolerance to abiotic stresses and into the processes involved in flowering and anthesis. The recently published *H. undatus* draft genome has paved the way for further molecular breeding programs.

Protocols for embryo rescue, androgenesis and gynogenesis, in situ autopolyploidization, and flow cytometry analysis were developed, paving the way for technical advances in other challenging plant species.

Finally, the increased research interest in *Hylocereus* – as an example of an underutilized crop – is reflected in the number of scientific publications over the years, which have risen from only one in 1994 to 77 and 64 in 2019 and 2020, respectively [85].

## Author details

French Associates Institute for Agriculture and Biotechnology of Drylands, Blaustein Institutes for Desert Research, Sede Boqer Campus, Ben-Gurion University of the Negev, P.O.B. 653, Beer Sheva 84104, Israel.
